# A Case of Very Late Onset Schizophrenia-Like Psychosis Presenting in a Patient Forty Years Post Acoustic Neuroma Resection

**DOI:** 10.1192/bjo.2025.10769

**Published:** 2025-06-20

**Authors:** Sita Shah, Emad Sidhom, Cristina Levinte, Eladia Ruiz-Mendoza, Julius Essem

**Affiliations:** Cambridge and Peterborough NHS Foundation Trust, Peterborough, United Kingdom

## Abstract

**Aims:** Very late onset schizophrenia-like psychosis (VLOSLP) can be defined as individuals presenting with symptoms of psychosis after the age of 60 that cannot be attributed to an “affective disorder or focal or progressive structural brain abnormality”. Despite being described by an international group consensus in 1998, this diagnosis is not included in ICD–11 or DSM–5 manuals.

**Methods:** A 79-year-old female presented with a 3-month history of auditory hallucinations, involving 10 voices talking in the 2nd and 3rd person and providing commands. The patient also described visual, tactile and olfactory hallucinations.

The patient did not have any previous psychiatric history. Significant past medical history included previous surgical removal of right-sided acoustic neuroma resulting in facial nerve palsy.

On assessment, there was right-sided facial paralysis, deafness and slurred speech. The patient was calm and well kempt. Speech and mood were normal. There was no formal thought disorder and the patient was not responding to unseen stimuli. They were orientated to time, person and place. The patient displayed insight into their mental state.

CT head showed mild small vessel disease. The patient scored 79/100 (attention 17/18, memory 16/26, fluency 8/14, language 23/26, visuospatial 15/16) on the Addenbrooke’s cognitive examination, whilst psychotic and without hearing aids in situ.

The patient was started on aripiprazole, titrated to a dose of 20 mg. Fewer voices were heard and became incredibly faint, with there being some days where she was unable to hear them. The patient had not been experiencing hallucinations at 3 months post-discharge.

**Results:** There have been some case reports of acoustic neuromas presenting with psychiatric symptoms such as hallucinations and persecutory delusions and emerging post resection.

Individuals with hearing impairment are significantly more likely to develop psychosis. Hearing impairment is a modifiable risk factor for developing dementia and individuals with VLOSLP display an increased risk of developing dementia. It has been postulated whether VLOSLP could be a prodrome for dementia. The mainstay of treatment for VLOSLP involves low dose atypical antipsychotics.

**Conclusion:** We describe a case of VLOSLP in a patient 40 years post acoustic neuroma removal. There needs to be further work to investigate neuropsychiatric presentations post acoustic neuroma removal. There is increasing evidence to suggest an association between hearing impairment and development of psychosis and dementia. Any hearing impairment should be treated promptly. Patients with VLOSLP should be monitored for the development of dementia given it could be a prodrome for dementia.

